# Compartmentalised cAMP signalling in the primary cilium

**DOI:** 10.3389/fphys.2023.1187134

**Published:** 2023-05-09

**Authors:** Ester Paolocci, Manuela Zaccolo

**Affiliations:** Department of Physiology, Anatomy and Genetics, The University of Oxford, Oxford, United Kingdom

**Keywords:** primary cilium, cAMP signaling, GPCR (G protein coupled receptor), autosomal dominanat polycystic kidney disease (ADPKD), FRET microscopy

## Abstract

cAMP is a universal second messenger that relies on precise spatio-temporal regulation to control varied, and often opposing, cellular functions. This is achieved via selective activation of effectors embedded in multiprotein complexes, or signalosomes, that reside at distinct subcellular locations. cAMP is also one of many pathways known to operate within the primary cilium. Dysfunction of ciliary signaling leads to a class of diseases known as ciliopathies. In Autosomal Dominant Polycystic Kidney Disease (ADPKD), a ciliopathy characterized by the formation of fluid-filled kidney cysts, upregulation of cAMP signaling is known to drive cystogenesis. For decades it has been debated whether the primary cilium is an independent cAMP sub-compartment, or whether it shares a diffusible pool of cAMP with the cell body. Recent studies now suggest it is a specific pool of cAMP generated in the cilium that propels cyst formation in ADPKD, supporting the notion that this antenna-like organelle is a compartment within which cAMP signaling occurs independently from cAMP signaling in the bulk cytosol. Here we present examples of cAMP function in the cilium which suggest this mysterious organelle is home to more than one cAMP signalosome. We review evidence that ciliary membrane localization of G-Protein Coupled Receptors (GPCRs) determines their downstream function and discuss how optogenetic tools have contributed to establish that cAMP generated in the primary cilium can drive cystogenesis.

## Introduction

### Compartmentalization of the cAMP pathway

The primary cilium has recently protruded from obscurity as an intriguing example of compartmentalized cAMP signaling. cAMP is a second messenger that translates signals from hormones, neurotransmitters, and other extracellular stimuli into a variety of intracellular responses, ranging from cell proliferation to apoptosis, from cell differentiation to regulation of metabolic functions. (Yamamoto et al., 1988; Weissinger et al., 1997; Stork and Schmitt, 2002). Such disparate outcomes are achieved through cAMP-dependent modulation of a limited number of effectors that, in turn, can impact a myriad of different targets, including channels, receptors, transporters and pumps, the cytoskeleton, the transcriptional machinery, metabolic enzymes and many others. Fidelity of signal transduction from a specific extracellular stimulus to the appropriate cellular function is achieved through tight spatio-temporal control of the cAMP signal. (Zaccolo et al., 2021). cAMP is synthesized from ATP by adenylyl cyclases (ACs). Activation of a G-protein coupled receptor (GPCR) by an extracellular signal (Levitzki, 1988; Mons et al., 1998; Selbie and Hill, 1998) leads to its association with a G protein, a trimeric αβγ complex, where the *α* subunit can either trigger (Gαs) or supress (Gαi) AC activity, resulting in increased or reduced cAMP synthesis, respectively (Rodbell et al., 1971). Mammalian cells express nine different membrane-integral AC isoforms (AC1-9) and one soluble isoform, AC10, that is not activated by Gαs but by bicarbonate (Dessauer et al., 2017; Wiggins et al., 2018). Once generated, cAMP binds to a few effector proteins: protein kinase A (PKA) (Walsh et al., 1968; Taylor et al., 2008), the exchange protein directly activated by cAMP (EPAC) (de Rooij et al., 1998), cyclic nucleotide-gated ion channels (Fesenko et al., 1985), and Popeye domain containing proteins (POPDC) (Brand, 2005). PKA, the most extensively studied cAMP effector, when inactive is a heterotetramer resulting from the association of a regulatory (R) dimer and two catalytic (C) subunits. (Walsh et al., 1968; Corbin and Keely, 1977; Taylor et al., 1993). Combinations of 4R subunits (RIα, RIβ, RIIα, and RIIβ) and 3C subunits (Cα, Cβ, and Cϒ) can make up the inactive PKA holoenzyme R2C2. (Diskar et al., 2010). When cAMP binds to the R subunits, a conformational change occurs, releasing the inhibitory effect of R over the 2C subunits, allowing the C subunits to phosphorylate protein targets (Kim et al., 2007; Taylor et al., 2013). Although for a long time the consensus has been that, upon cAMP binding to R subunits the C subunit dissociates from the holoenzyme to then go on to phosphorylate target proteins, the concept of a freely diffusing C subunit is at odds with the model of compartmentalisation. More recently, alternative hypotheses have been put forwards to explain how the effects of active PKA may remain local (Chao et al., 2019). In one model, target phosphorylation does not require C dissociation from R subunit but binding of cAMP is sufficient to induce a conformational change that exposes the catalytic pocket in C which can then phosphorylate targets within a range of about 15–25 nm (Smith et al., 2017). An alternative hypothesis is that cells express a significant excess of R subunits, that are therefore not coupled with C. On activation of PKA, C is released but can quickly be recaptured by free R subunits that are present nearby. (Walker-Gray et al., 2017).

cAMP is hydrolysed by phosphodiesterases (PDEs). PDEs are a super-family of enzymes that includes 11 families (PDE1-11). Of these, PDE4, PDE7, and PDE8 selectively hydrolyse cAMP. PDE1, PDE2, PDE3, PDE10, and PDE11 hydrolyse both cAMP and cGMP, whereas PDE5, PDE6, and PDE9 are cGMP selective. Several families of PDEs include multiple genes and each gene can generate differently spliced transcripts or be transcribed from different initiation sites, resulting in a large number of PDE isoforms. These isoforms display different affinities for cAMP and enzyme kinetics, their activity can be regulated by different mechanisms (e.g., phosphorylation, Ca^2+^ or cGMP binding) and individual isoforms localise to specific subcellular sites, providing a means to generate subcellular cAMP gradients, or confined cAMP pools, via differential cAMP degradation at different locations. (Marchmont and Houslay, 1980; Conti et al., 1984; Jurevicius and Fischmeister, 1996; Mongillo et al., 2004; Bender and Beavo, 2006; Lugnier, 2006; Di Benedetto et al., 2008). In addition to the PDEs, the cAMP signal mediated by PKA, can also be quenched by phosphatases (PPs), which counteract PKA phosphorylation of target proteins (Ingebritsen and Cohen, 1983; Francis et al., 2011). A further class of proteins that contributes to compartmentalise the cAMP signal are the A-kinase Anchoring Proteins (AKAPs) which dock PKA to specific cellular locations where proteins that are phosphorylated by PKA are also localised. As a result, when the AKAP-tethered PKA is activated by cAMP, it preferentially phosphorylates the nearby target. In addition to PKA and its targets, AKAPs can also bind other signaling molecules, thus assembling signaling platforms, or signalosomes, where the cAMP signal can be locally generated (e.g., through AKAP association with GPCR or ACs), delivered (via tethered of PKA or EPAC) and regulated (through association of AKAPs with PDEs, PPs, other kinases) (Colledge and Scott, 1999; Wong and Scott, 2004).

### The primary cilium

Due to the compartmentalised nature of cAMP signaling, it is no surprise that often organelles are the sites where cAMP signalosomes are found. (Langeberg and Scott, 2015; Monterisi and Zaccolo, 2017; Torres-Quesada et al., 2017). The primary cilium, an organelle that had long been ignored since it was first discovered in 1898 by Zimmerman, (Zimmermann, 1898), has more recently garnered much attention due to the fact that mutations in ciliary proteins lead to a family of diseases known as “ciliopathies”, which are characterised by developmental defects, sensory malfunction, obesity, reproductive problems, and cysts of the liver and kidneys (Hildebrandt et al., 2011). The primary cilium is a thin projection that extends from the surface of most mammalian cells, consisting of a microtubule-based axoneme and a surrounding membrane. The cilium is anchored to the cell by a basal body, which is derived from the centrosome. This antenna-like organelle plays a vital role in mechanosensory functions (Bangs and Anderson, 2017; Kiesel et al., 2020; Ho and Stearns, 2021), such as fluid-flow sensing, and is important for photoreception (Bujakowska et al., 2017), and olfaction (Doroquez et al., 2014). The cilium is also a hub for cell signaling, hosting a variety of pathways including Sonic Hedgehog, Wnt, Notch, Hippo, PDGF, MTOR, TGF-beta, Ca^2+^ and cAMP signaling (Wheway et al., 2018). In the first identified ciliopathy, Autosomal Dominant Polycystic Kidney Disease (ADPKD), excessive cAMP signaling was shown to drive cystogenesis. ADPKD is caused by mutations in either Polycystin 1 (PC1) or Polycystin 2 (PC2), two ciliary proteins which regulate intracellular and ciliary Ca^2+^ signaling. cAMP and Ca^2+^ signaling were demonstrated to work hand-in hand to elicit intracellular and ciliary responses, but there has been much debate on whether the primary cilium harbors its own pool of cAMP, independent of cytosolic cAMP, or whether this second messenger freely diffuses between the organelle and the cell body. Recent research supports the notion that it is ciliary cAMP which drives cystogenesis in ADPKD and that ciliary cAMP signaling controls Hedgehog transcription. (Moore et al., 2016; Hansen et al., 2022). A functionally independent pool of ciliary cAMP begs the question of whether specific cAMP signalosomes exist within the cilium, especially since the primary cilium and the cAMP pathway are involved in a variety of intracellular processes. Below we discuss the evidence in support of multiple cAMP subdomains being present within the cilium and centrosomes, we explore the role of cAMP compartmentalisation in the context of ADPKD and we discuss the technology that has been used, and could be further developed, to detect compartmentalisation of the cAMP signal within the organelle.

### cAMP signalosomes in the primary cilium

To understand how the primary cilium could function as a compartmentalized cAMP microenvironment, it is important to consider the structure of the primary cilium. The organelle is highly conserved through eukaryotic evolution and is found from *Chlamydomonas* and *Tetrahymena* to worms, flies, mice and humans. (Hilgendorf et al., 2016). The axoneme of the cilium extends from the basal body, which forms from the mother centriole of most cell types during cell cycle arrest. The basal body is constituted by a group of specialized proteins which function as a platform for cilia growth. (Satir and Christensen, 2007). A transition zone (TZ) separates the ciliary sub-domain from the cytosol, strictly gating the proteins of the ciliary compartment (Reiter et al., 2012). Mutations in proteins of the transition zone lead to ciliopathies, such as Meckel-Gruber Syndrome (MKS), Joubert Syndrome (JBTS), Bardet-Biedl Syndrome (BBS), and Nephronophthisis (NPHP) (Reiter et al., 2012). The ciliary membrane differs from the plasma membrane and is constituted of a unique bilayer where the amount and localization of distinct phosphoinositide lipid species is defined by opposing actions of lipid kinases and phosphatases (Conduit and Vanhaesebroeck, 2020). Aside from its intricate structure, the ciliary membrane also hosts a plethora of receptors, including GPCRs (Hilgendorf et al., 2016) and signaling pathways that operate in concert with cAMP signaling. Furthermore, since the surface to volume ratio of the cilium is 13-fold higher than that of the cell body, (Arena and Hofer, 2021), if G_αs_-coupled GPCRs have a similar density on ciliary and cell membranes, then the higher surface to volume ratio of the primary cilium should make the organelle a cAMP hotspot. A ciliary pool of cAMP has been shown to regulate the best characterized signaling pathway within the cilium, the Sonic Hedgehog (SHh) pathway (Bangs and Anderson, 2017).

### cAMP and regulation of hedgehog activity

**FIGURE 1:**
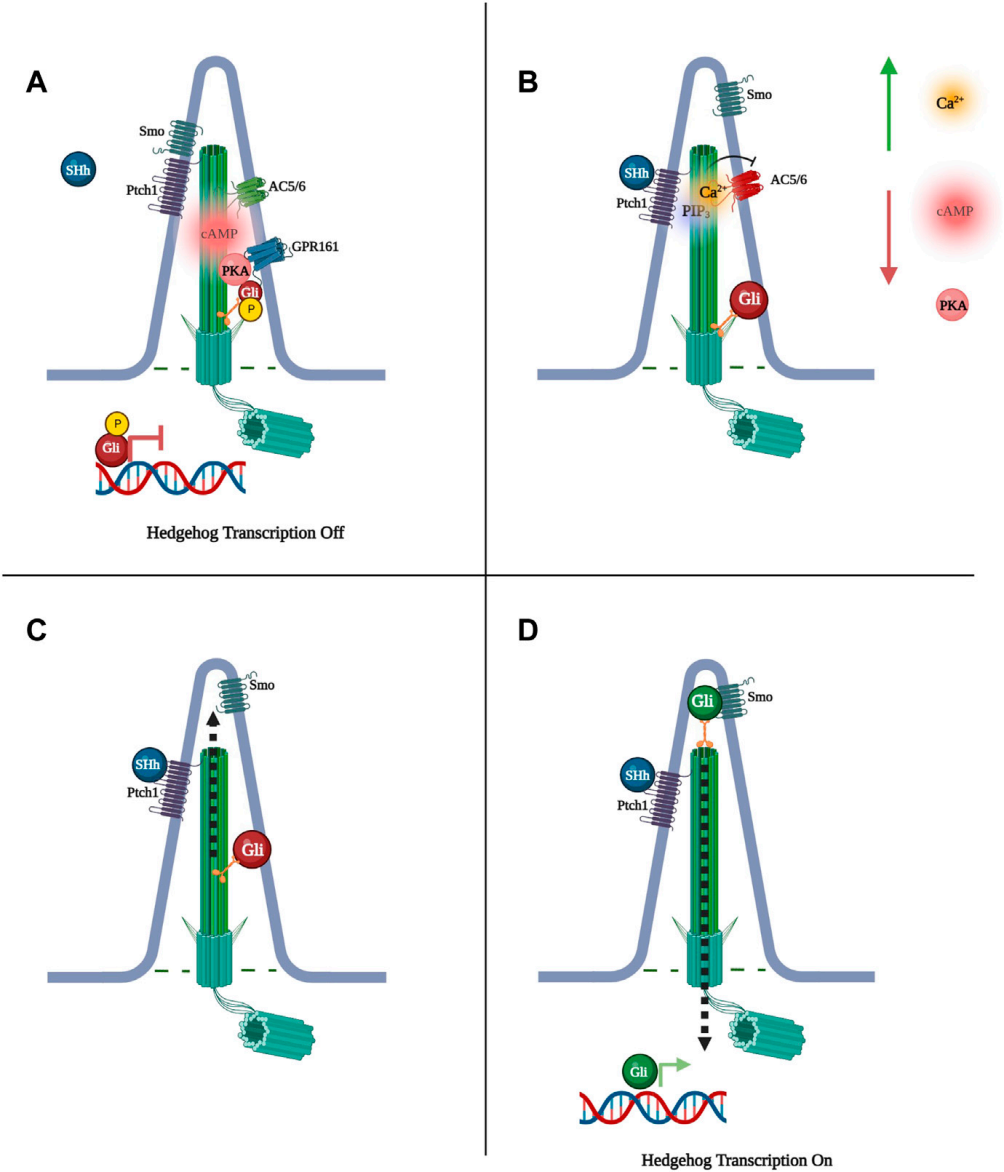
Sonic Hedgehog (SHh) signaling is regulated by Protein Kinase A (PKA) phosphorylation of glioma-associated oncogene (Gli) transcription factors. **(A)** When PKA phosphorylates Gli, Gli cannot be processed by Smo into its active state and SHh-dependent transcription does not occur. **(B)** Binding of Shh to Pitch1 activates phosphatidylinositol (3,4,5)-triphosphate (PIP3), increasing ciliary Ca^2+^ levels and preventing cAMP synthesis via Ca^2+^-inhibited adenylyl cyclase 5 and 6 (AC5/6), subsequently decreasing PKA phosphorylation of Gli. **(C)** Gli moves to the tip of the primary cilium. **(D)** Smoothened (Smo) is freed to activate Gli, Gli exits the cilium to initiate transcription.

Hedgehog signaling works as a mitogen, driving important functions such as growth and morphogenesis in a variety of vertebrate tissues, particularly the nervous system, limbs, and skeleton (Chiang et al., 1996). In the absence of the SHh ligand, the SHh receptor patched 1 (Ptch1) represses the activator smoothened (Smo), inhibiting the downstream pathway. (Figure 1). Binding of one of three ligands, Sonic, Indian, or Desert Hedgehog to Ptch1, releases Smo from Ptch1, allowing Smo to mediate splicing of activating forms of glioma-associated oncogene (Gli) transcription factors. (Ho and Stearns, 2021) (Figure 1). Primary cilia play three different roles in Hedgehog signaling. Firstly, as the location where the Ptch1 receptor is concentrated, the cilium is the site of initiation of Hedgehog signaling. Secondly, Ptch1 repression of Smo occurs in the cilium, and when signaling is activated, Smo accumulates in the ciliary membrane (Corbit et al., 2005). Thirdly, the primary cilium is necessary for the processing of Gli2 and Gli3, as these transcription factors travel via Intra Flagellar Transport (IFT) to the top of the cilia, where they are cleaved into activator or repressor forms, translating the SHh signal into changes in cellular function (Huangfu et al., 2003; Haycraft et al., 2005; Ho and Stearns, 2021). The hedgehog pathway is known to be regulated by cAMP signaling through a negative feedback loop, orchestrated at the base of the cilium, whereby PKA phosphorylation of Gli2 restrains its activation (Tuson et al., 2011). In response, SHh reduces ciliary cAMP and PKA activity through regulation of phosphatidylinositol (3,4,5)-triphosphate (PIP3) and consequent Ca^2+^ signaling, which inhibits AC5 and AC6 and cAMP synthesis (Moore et al., 2016). Such a balanced mechanism between cAMP and Hedgehog would require tight spatio-temporal control of both pathways, and would therefore suggest a ciliary cAMP signalosome is present in the cilium to direct this process.

Studies have identified the orphan receptor of the rhodopsin GPCR family, Gpr161, to localize to the cilium, where its transport is regulated by the tubby family protein Tulp3 and IFT-A. (Mukhopadhyay et al., 2013). Constitutive activity of Gpr161 increases cAMP in the cilium, altering Gli3 to its repressor form, resulting in suppression of SHh signaling. PKA phosphorylation of Gli at the base of the cilium leads to Gli repression, suggesting that Gpr161 works through cAMP and subsequent PKA activation to silence SHh signaling (Huangfu et al., 2003; Haycraft et al., 2005; Ho and Stearns, 2021). Furthermore, SHh signaling results in Gpr161 exit from the cilium, suppressing receptor signaling, while loss of Gpr161 leads to SHh hyperactivity, supporting a feedback loop between Gpr161 and SHh. (Mukhopadhyay et al., 2013). Research in zebrafish also identified Gpr161 as a high-affinity AKAP for PKA RI whereby RI binds the hydrophobic protein-protein interaction interface in the cytoplasmic terminal tail of Gpr161. This particular study showed that the RI-Gpr161 complex localizes to the plasma membrane but, on overexpression, RI and Gpr161 were shown to also interact at the primary cilium. A L465 mutation in Gpr161, which disrupts Gpr161 interaction with PKA, led to significantly reduced ciliary localization of RIα-GFP, suggesting Gpr161 functions at the cilium as an AKAP for PKA. (Bachmann et al., 2016; Pal et al., 2016). Interestingly, in primary hippocampal neurons Gpr161 has been suggested to couple with the calcium activated AC3 (Mukhopadhyay et al., 2013), potentially constituting a signalosome that is distinct from the Ptch1/PIP3/AC5/6, and undergoes opposing regulation by calcium. The endogenous ligand for Gpr161 remains unknown.

Another orphan receptor that has been identified in the primary cilium, Gpr175, was shown to interact with ciliary Gαi to decrease cAMP levels and PKA activity, modulating the formation of Gli repressor. However, Gpr175 depletion only led to a small reduction in SHh signalling, suggesting that Gpr175 plays a regulatory, rather than an essential and direct role in the SHh pathway (Singh et al., 2015). Based on these studies, at least one compartmentalised cAMP signal appears to exist in the cilium where it regulates Hedgehog transcription. Whether Gpr161 and Gpr175 are part of the same or independent signalosome is currently unknown.

### cAMP signaling and cilium length

The length of the primary cilium has been investigated in relation to ciliopathies such as ADPKD, however it is uncertain whether increased cilia length aggravates disease progression or whether it functions as a compensatory mechanism. The general view is that altered ciliary length in either direction is detrimental to cellular homeostasis (Scarinci et al., 2022). To complicate things further, cAMP appears to contribute both to ciliary growth and retraction, suggesting that more than one cAMP signalosome exists in the cilium to regulate the organelle’s length.

**FIGURE 2:**
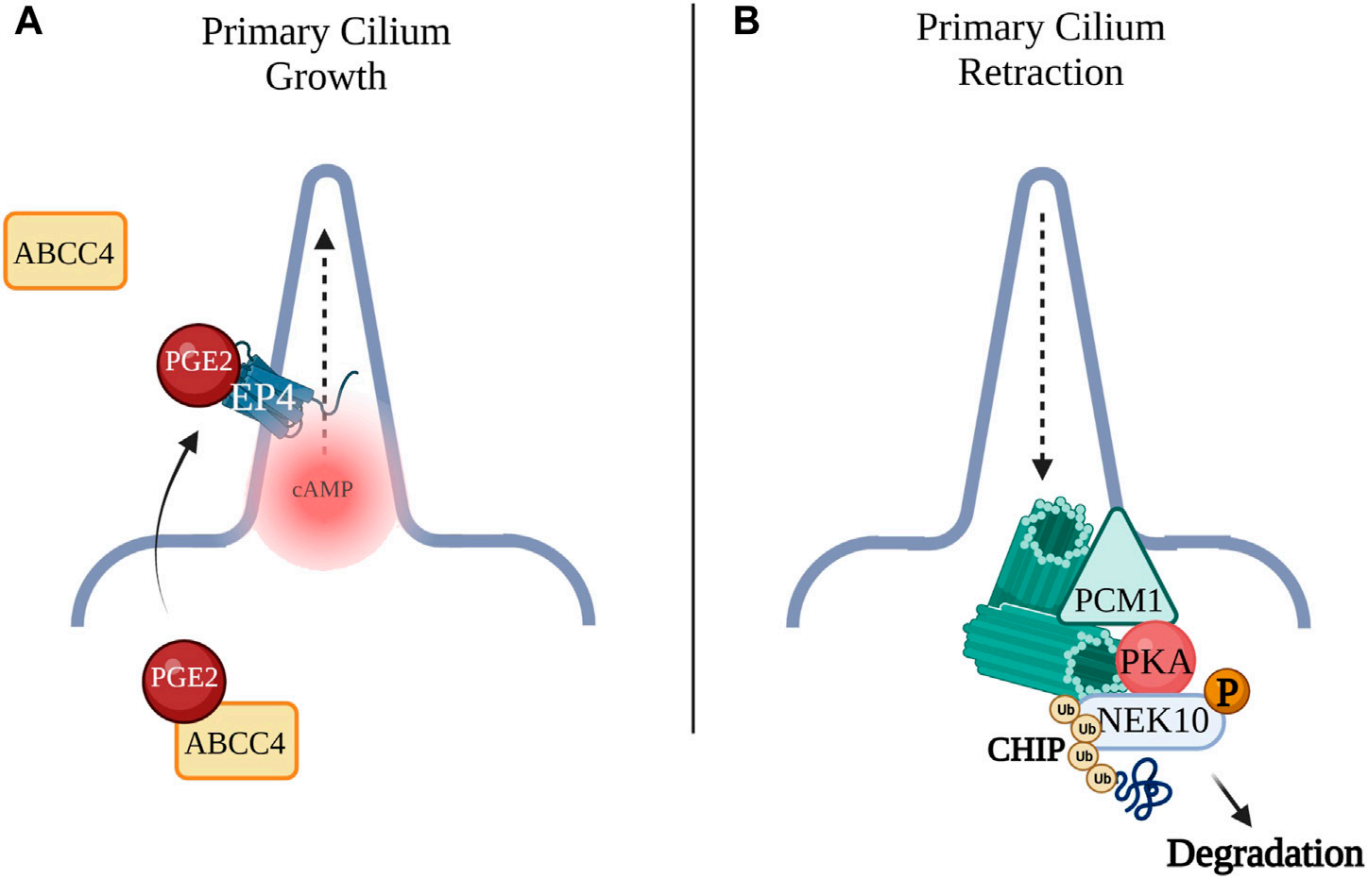
**(A)** Prostaglandin E2 (PGE2) is exported by the ATP-binding cassette transporter protein (ABCC4) across the plasma membrane to the extracellular space where PGE2 can bind the G_αs_-coupled receptor, EP4 to promote cilia formation as well as elongation. **(B)** Pericentriolar material 1 (PCM1) acts as an AKAP for PKA. GPCR activation of PKA leads to phosphorylation of NEK10, a kinase required for ciliary biogenesis. Phosphorylation of NIMA-related Kinase 10 (NEK10) by PKA leads to ubiquitination and proteolysis of the kinase by E3 ligase CHIP leading to cilia retraction.

A number of studies support a role of cAMP in primary cilium elongation. For example, Prostaglandin E2 (PGE2) and its G_αs_-coupled receptor, EP4, were shown to promote cilia formation as well as elongation (Figure 2). PGE2 is exported from the cell to the extracellular space to bind the EP receptor on the cilium membrane. In Zebrafish the leakytail (lkt) mutation, which affects the ATP-binding cassette transporter protein (ABCC4) responsible for the export of PGE2, shows ciliogenesis deficits (Jin and Zhong, 2022), suggesting the EP4 receptor interacts with specific ciliary ACs to generate cAMP and thus regulate organelle growth.

Another study demonstrated that cAMP and PKA regulate both cilia length and function in vascular endothelial cells. In these cells, activation of PKA significantly increased cilia length through a pathway involving cofilin and protein phosphatase-1 (PP-1)-mediated restructuring of actin stress fibers into cortical filamentous actin. The change in cilia length upon cAMP upregulation also led to enhanced flow sensing activity (Abdul-Majeed et al., 2012).

Consistent with a role of cAMP in promoting cilia length, higher levels of the second messenger are found in cystic tissue from ADPKD patients where the primary cilia of renal cells are abnormally elongated, (Shao et al., 2020), although in the renal ciliopathy it is unclear whether elongated cilia are a result of increased levels of cAMP, or whether altered signaling of a longer cilium leads to enhanced cAMP signaling. Narrowing in on specific ciliary cAMP signalosomes affecting cilium length could help explain why these structural ciliary patterns are observed, and targeting such signalosomes could potentially be a therapeutic strategy for ciliopathies such as ADPKD.

The cAMP pathway not only promotes cilium elongation, but it also appears to drive cilia retraction, thus regulating two opposing cellular functions. The primary cilium and the centrosome are interdependent and are regulated by a complex network of PKA phosphorylation targets, suggesting that distinct cAMP signalosomes may function to control cilia retraction. Primary cilium assembly is induced when cells leave the cell cycle to enter G_0_ and ciliary vesicles begin to dock at distal sites of the basal body (Porpora et al., 2018). Axonemal microtubules grow and when the nascent cilium fuses with the plasma membrane, it matures (Sánchez and Dynlacht, 2016). The pericentriolar matrix, which acts as a scaffold for ciliogenesis, is constituted by multiple proteins, including pericentriolar material 1 (PCM1). PCM1 depletion has been shown to lead to loss of primary cilia and PCM1 interaction with CEP290 and Bardet-Biedl Syndrome (BBS) proteins are required for ciliogenesis (Kim et al., 2008). CEP290 and PCM1, together, recruit to the cilium Rab8, a small GTPase which functions with the BBS proteins to promote ciliogenesis (Kim et al., 2008). Of note, PCM1 has also been identified as an AKAP that anchors PKA at the centrosome. In addition, GPCR activation of PKA leads to phosphorylation of NIMA-related Kinase 10 (NEK10), a kinase required for ciliary biogenesis in both mammals and lower vertebrates. Phosphorylation of NEK10 by PKA leads to ubiquitination and proteolysis of the kinase by E3 ligase CHIP Figure 2. This suggests that a cAMP signalosome, with PCM1 possibly operating as an AKAP, exists at the centrosomes where it coordinates cilia resorption. (Porpora et al., 2018). Loss of cilia due to aberrant PKA/NEK10/CHIP function was demonstrated in several ciliopathies and cancers such as glioblastoma, astrocytoma, and high-grade prostate cancer (Valente et al., 2014; Porpora et al., 2018).

The PKA/NEK10/CHIP structure is not the only cAMP complex reported at the centrosome. Senatore et al. (2021) used several models, including Medaka fish, to demonstrate that PKA phosphorylation of the Orofacial Digital Type 1 (OFD1) protein coordinates OFD1 proteolysis at the centrosome through the E3 ubiquitin ligase praja2 and the ubiquituin-proteasome system (UPS). In line with these findings, non-phosphorylatable OFD1 mutants were shown to markedly change cilia morphology and dynamics. The impaired ciliogenesis observed in the Medaka fish upon disruption of this signaling axis was found to lead to developmental defects. This data is consistent with the phenotype observed in the OFD1 syndrome, an X-linked congenital ciliopathy which manifests with facial, oral cavity and digital malformations and, in the majority of cases, with polycystic kidney disease. (Senatore et al., 2021). In another study, AC3 stimulation, believed to originate from the primary cilium, led to activation of PKA at the centrosomes of embryonic, postnatal and adult migrating neurons, hinting at a further cAMP signalosome linking the two interdependent organelles. In these neurons, stimulation of centrosomal PKA was demonstrated to regulate centrosome organization, nucleokinesis as well as cell migration (Stoufflet et al., 2020). Whether AC3 forms a complex with PCM1, functioning as an AKAP at the centrosome, and whether AC3 activation leads to downstream phosphorylation of OFD1 or NEK10, is not known. Data however support cAMP compartmentalization at the centrosomes, where the second messenger not only regulates ciliogenesis and cilium retraction, but modulates the cell cycle (Terrin et al., 2012). Excessive cell proliferation is not only a key aspect of ADPKD, but also a prominent driver of cancer and targeting the cAMP signalosome regulating cell cycle progression could help tackle an extensive number of pathological conditions.

### GPCR signalling in the cilium

In addition to Gpr161 and Gpr175 discussed above, the primary cilium hosts multiple other GPCRs. Several studies show that the same GPCR can localise to the cilium or to the plasma membrane but achieves distinct outcomes depending on its localisation. For example, the primary cilia of cholangiocytes are mechano-, chemo- and osmosensory organelles that also control proliferation of these epithelial cells. (Masyuk et al., 2006; Masyuk et al. 2008; Masyuk et al. 2013). Cholangiocytes line bile ducts, where they transport bile acid and secrete bicarbonate (Tabibian et al., 2013). Bile acid signalling is transmitted through TGR5, a GPCR which localises to the plasma membrane, the subapical compartment and the cilium (Keitel and Häussinger, 2011; Keitel and Häussinger, 2012; Masyuk et al., 2015). The presence or absence of cilia in cultured cholangiocytes determines the impact of TGR5 agonists. (Masyuk, Masyuk, and LaRusso 2015) In non-ciliated cholangiocytes, TGR5 show marked colocalization with Gαs and its activation leads to elevation in cAMP, ERK pathway inhibition and increased cell proliferation. On the other hand, in ciliated cholangiocytes TGR5 couples to Gαi, and agonist stimulation leads to decreased cAMP, activation of ERK, and reduced cell proliferation (Masyuk et al., 2015). These findings suggest that TGR5 is functionally paired to Gαs at the plasma membrane, but interacts with Gαi when in the cilium.

**FIGURE 3:**
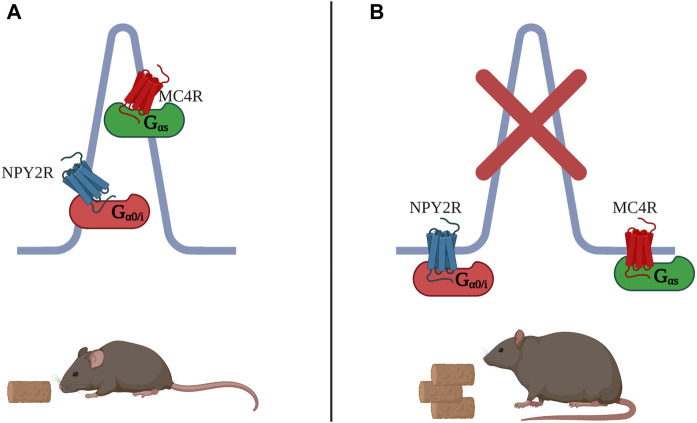
**(A)** MC4R is a G_αs-_coupled receptor which localizes to the primary cilium of hypothalamus neurons to control food intake. Also in cilia of hypothalamus neurons, NPY2R regulates food intake, but in contrast to MC4R, it does so through decrease in cAMP, as NPY2R is G_αi-_coupled. **(B)** Both MC4R and NPY2R, when excluded from the cilium, fail to repress food intake. Many ciliopathies as well as complete loss of primary cilia lead to obesity.

Other GPCRs may reside in the same primary cilium to control the same process via different signalling mechanisms. For example, Melanocortin-4 Receptor (MC4R) is a GPCR involved in food consumption and mutations in this receptor are the most common cause of monogenic obesity (Vaisse et al., 1998; Vaisse et al., 2000; Lubrano-Berthelier et al., 2006). MC4R was recently reported to localise to the primary cilium and a genetic approach showed that ciliary localisation of MCR4 in neurons is required for proper MC4R Gαs signalling (Walker-Gray et al., 2017). Genetic knock out (KO) of intraflagellar transport protein 88 (lft88) ablated primary cilia in mice and proved cilia are necessary for pharmacological activation of MC4R and subsequent anorexigenic effect. Cilium removal in MC4R-expressing cells from the mouse hypothalamus phenocopied germline loss of MC4R, which is characterised by obesity. Since MCR4 activation leads to AC stimulation, the study also tested whether inhibiting ACs in the cilia of MC4R-expressing neurons had consequences on food intake. Indeed, inhibition of ciliary ACs was achieved by expressing the constitutively active Gαi-coupled receptor, GPR88, exclusively in the cilium. Silencing of AC’s in the organelle caused hyperphagia and obesity. The authors propose that defects in ciliary localisation of MC4R lead to loss of receptor sensitivity, inherited obesity syndromes and accounts for the high rates of obesity found in other ciliopathies (Patel, 1999; Wang et al., 2021). Furthermore, genetically modified mice which cannot transport NPY2R, another GPCR controlling food intake, in neuronal cilia, but normally express the receptor at the plasma membrane, develop an obese phenotype and are insensitive to the anorexigenic ligand PYY3-36, indicating that NPY2R ciliary localisation is vital for ligand-dependant signalling to control food intake (Loktev and Jackson, 2013). Just like MC4R, NPY2R is found in cilia of hypothalamus neurons. Although there is no clear evidence that the two receptors reside in the same cilium, both receptors require ciliary localisation to mediate the same function within the same brain region. Interestingly, NPY2R is coupled to G0/Gi, and thus its activation reduce cAMP, while MC4R is Gαs coupled and its activation increases ciliary cAMP (Figure 3). The contrasting signalling modality of these receptors hints at two separate ciliary signalosomes with different signaling components, potentially localizing within one cilium, working to balance the same process.

Kisspeptin is another GPCR localised to the cilium in gonadotropin-releasing hormone (GnRH) secreting neurons. Kisspeptin signalling is involved in the onset of puberty and mature reproductive function (Koemeter-Cox et al., 2014). In adult animals, multi-ciliated GnRH neurons have several KISS1R-positive primary cilia, and the percentage of these multi-ciliated neurons increases with postnatal and sexual development. Disruption of these cilia leads to a marked decrease in kisspeptin-mediated GnRH action (Koemeter-Cox et al., 2014). Recently, the β-adrenergic receptor (β2AR) has also been shown to localise to neuronal cilia in mouse hippocampus (Yao et al., 2016) where its activation contributes to hippocampal synaptic plasticity (Hagena et al., 2016). Of note, β2AR colocalises in neuronal cilia with the non-selective cation channel polycystic kidney disease 2-like 1 (Pkd2l1). Mice lacking Pkd2l1 have disrupted β2AR ciliary localisation, decreased cAMP in the brain, and higher rates of pentylenetetrazol-induced seizures (Yao et al., 2016). The hypothesis is that the Gαs coupled β2AR forms a complex at the cilium with Pkd2l1 to increase cAMP signalling and inhibit neuronal excitability (Yao et al., 2016).

TGR5, MC4R, NPY2R, β2AR and Kisspeptin are examples of how the same GPCR at the cilium can result in one outcome while a different response is triggered when the same receptor operates from the plasma membrane. How the primary cilium can achieve such feats is still a mystery. One hypothesis would look to the cilium as a highly compartmentalised organelle. Perhaps the complexes that interact with the receptor, such as AKAPs and G-proteins, are distinct at the two locations. Further, the downstream effectors of GPCRs and cAMP at the cilium could be different to effectors localised in the cell body. GPCR in the cilium could also be in closer proximity to downstream functional components and a temporal aspect could be at play whereby cilium elements could be engaged more rapidly than those in the cytosol. Finally, enhanced isolation of the cAMP signal, due to the primary cilium structure, might likewise contribute to the distinct function of these GPCRs. Whatever explanation turns out to be correct, it is likely that compartmentalisation of cAMP at the cilium plays a role.

### Ciliary cAMP signalosomes and ADPKD

**FIGURE 4:**
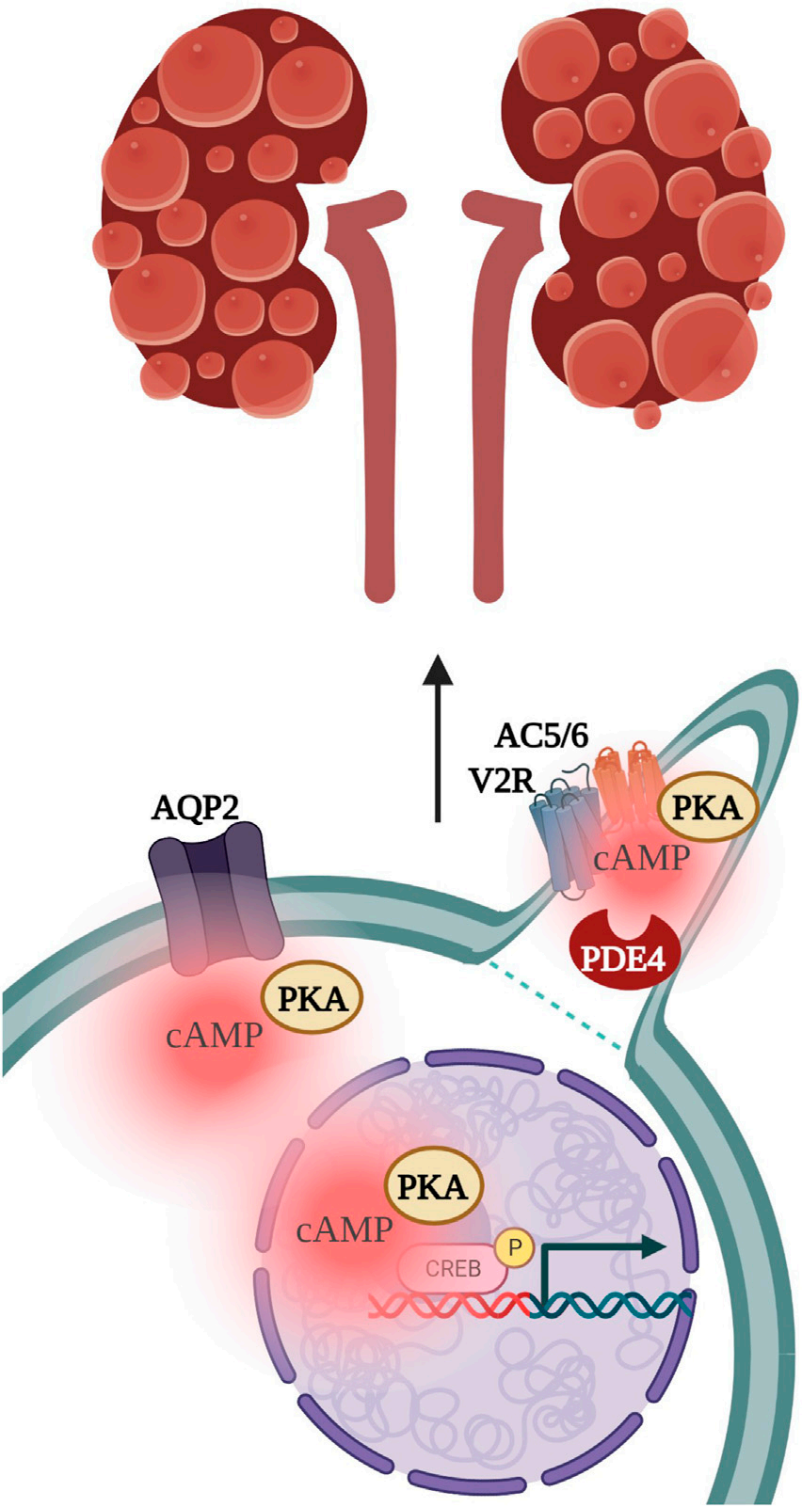
Vasopressin stimulation of the vasopressin type 2 receptor (V2R) localised to the primary cilium activates PKA-dependent phosphorylation of Aquaporin 2 (AQP2) channels and of the transcription factor cAMP-response element binding protein (CREB), to subsequently drive kidney cystogenesis. PKA, protein kinase A; AC5/6, adenylyl cyclase 5 and 6; PDE4, phosphodiesterase 4.

ADPKD has been linked with multiprotein complexes that could operate as cAMP signalosomes. The vasopressin type 2 receptor (V2R) localises to the primary cilium of the epithelial-like pig kidney cell line (LLC-PK) where it couples with Ca^2+^ inhibited AC5/AC6 (Figure 4). (Masyuk et al., 2006; Raychowdhury et al., 2009; Chien et al., 2010) Stimulation of V2R with Arginine-Vasopressin (AVP), in the presence of ATP, activates cation-selective channels in the ciliary membrane, to similar levels as addition of the non-specific adenylyl cyclase activator, forskolin. PKA inhibition after V2R stimulation reduced cation channel activity, suggesting V2R signals through ciliary cAMP synthesis for downstream effects such as PKA phosphorylation of aquaporin 2 (AQP2) and the transcription factor cAMP-response element binding protein (CREB) (Raychowdhury et al., 2009). Vasopressin levels are elevated in animal models as well as in patients affected by ADPKD (Meijer et al., 2011; Sherpa et al., 2019). Dysregulation of the V2R-generated cAMP pool in ADPKD is believed to influence fluid secretion and cell proliferation, which promotes cystogenesis (Cassola et al., 1993; Meijer et al., 2011; Reif et al., 2011; Rinschen et al., 2014). Consistently, Tolvaptan, a highly selective V2R antagonist approved for the treatment of ADPKD, has been shown to significantly reduce kidney volume and renal function decline in phase three clinical trials (Cassola et al., 1993).

Another set of proteins has been shown to form a complex with AC5 and AC6 in the primary cilium. Choi et al. found AC5/AC6 to interact with PDE4C, AKAP150, PKA, as well as Polycystin 1 and Polycystin-2 (Choi et al., 2011). As aforementioned, in ADPKD, a decrease in ciliary Ca^2+^, due to loss of PC1 or PC2 function activates AC5/6, increasing intracellular cAMP and driving cystogenesis (Choi et al., 2011; Rees et al., 2014; Spirli et al., 2017; Wang et al., 2018). The link between V2R and the PDE4C/AKAP150/PKA complex has not yet been made, but it is possible V2R couples with AC5/6 at AKAP150 to generate a pool of cAMP which functions in a feedback loop with polycystin-regulated Ca^2+^ activity, and which is controlled by PDE4C function. A way to confirm whether such a hypothesis is correct would be to measure cAMP using a Fluorescence resonance energy transfer (FRET) sensor localising to the AKAP150 complex in the primary cilium. In the final section we will discuss the evolution of imaging techniques that have been used to study cAMP signalling in the primary cilium.

## Studying cAMP signalling in the primary cilium with optogenetic tools

### FRET imaging of cAMP reporters

Ever since “ciliopathies” such as ADPKD were recognised as a severe class of diseases caused by dysfunction of the primary cilium, understanding ciliary signalling, including cAMP signalling, emerged as a priority. However, measuring cAMP signalling within the cilium, or even imaging the organelle, with its inherent reactional movements and transient nature, is challenging. FRET-based reporters for cAMP have been developed by several groups to narrow in on GPCR signalling in the cilium and, while some findings support the notion that the cilium is an independent cAMP subdomain, others serve to reject this hypothesis. Jiang et al. (2019) developed targeted sensors to measure cAMP in the primary cilium by fusing the EPAC-based sensors EPAC-H187 or EPAC-H188 to Arl13B, a protein exclusively localised to the cilium, (Duldulao et al., 2009), generating Arl13B-H187 and Arl13B-H188. No difference was found in ciliary versus cytosolic cAMP with either Arl13B-H187 or Arl13B-H188 in Inner Medullary Collecting Duct (IMCD3) cells. The authors also investigated which GPCRs are active in the cilium and found that, when overexpressed in IMCD3, V2R is primarily localised at the plasma membrane but vasopressin stimulation led to a sustained cAMP response of equal magnitude in the primary cilium and in the cytosol (Jiang et al., 2019). This finding suggests that cAMP synthesized in response to V2R stimulation at the plasma membrane leads to diffusion of cAMP through the transition zone into the cilium.

The cAMP response to the serotonin receptor, 5-hydroxytryptamine (5-HT6R), was also measured using several different FRET sensors with somewhat contrasting results. In one study, 5HT6 was found to localise to the cilium of primary neuronal cells. Over-expression of the serotonin receptor in the cilium of primary neurons elongated the organelle and increased dendritic branching, with inhibition of the GPCR causing the reverse phenotype. Rescue of 5HT6 in 5HT6R-KO mice was enough to increase cilia length and dendritic expansion, but primarily in neurons where 5HT6 localised exclusively to the cilium rather than also to the plasma membrane (Lesiak et al., 2018). The same study investigated a possible link between enhanced cAMP signalling upon 5-HT6R stimulation and increased cilia length but failed to see changes in cAMP signalling upon receptor stimulation (Lesiak et al., 2018). (Jiang et al. (2019) also set out to measure cAMP signalling upon 5HT6 activation. In their experiments, recombinant mCherry- 5-HT6R showed exclusive localisation to the cilium in some cells, while in others it was found both in the cilium and at the plasma membrane. The latter cells responded to serotonin stimulation with increased cAMP levels both in the cilium and in the cytosol, but cells with ciliary localisation of mCherry- 5-HT6R showed no cAMP response to serotonin. The same result was observed when the FRET sensor was directly fused to 5-HT6R (5-HT6 -EpacH187) (Jiang et al., 2019). These results are in line with previous studies from Lesiak et al. (2018) showing that GPCR activity was only observed after treating cells with ciliary localisation of 5HT6R with Smoothened agonist (SAG) but not without prior treatment, hinting that without SHh signalling there is no Gαs activity of 5HT6R at the cilium (Chaumont-Dubel et al., 2020). In a very elegant follow-up study by Jiang et al. (2019), IMCD3 overexpressing 5-HT6R and treated with digitonin, which results in permeabilization of the plasma membrane but not of the ciliary membrane, showed no cAMP response in the cell body or the cilium on application of 5-HT, indicating lack of 5-HT6R function in the cilium. However, stimulation of the same cells with the non-specific cAMP activator, forskolin, led to a small but reproducible cAMP response in the cilium but not the cytosol, indicating that the primary cilium is capable of generating its own cAMP when GPCR function is bypassed. In other cell types, e.g., 3T3 and mouse embryonic fibroblasts (MEFs), stimulation of endogenous 5-HT6R did produce a detectable increase in cAMP in the cilium, which was greatly enhanced when cells were pre-treated with SAG.

Overall, the findings of Jiang et al. delineate GPCR function in the cilium, but fail to observe a robust difference between the cAMP response at the cilium and the cell body or between the basal cAMP levels in the two compartments. In contrast, Moore et al. generated a PKA activity sensor targeted to the cilium via fusion of 5-HT6R to AKAR4, a FRET-based PKA activity reporter (Depry and Zhang, 2011), and in MEFs showed that PKA activity within the cilium is markedly higher than in the cytosol (Moore et al., 2016). In line with this finding, basal levels of cAMP were determined using the cAMP Difference Detector *in situ* (cADDis) reporters (Tewson et al., 2016) and mCherry-cADDis in the cell body reported 800 nM basal cAMP whereas 5-HT6 -mCherry-cADDis in the cilium reported 4 μM basal cAMP. Furthermore, after siRNA knock down (KD) of AC5/AC6 in IMCD3, 5-HT6 -mCherry-cADDis detected decreased levels of basal ciliary cAMP, confirming AC5/AC6 are responsible for cAMP production in the cilium. Knock down of Gαs in MEFs was confirmed with β2AR stimulation completely inhibiting cAMP signaling in the cilium. However, Gαs KD did not affect basal ciliary cAMP levels, mirroring Jiang’s findings that ACs function independently to GPCR signalling in the cilium for basal cAMP generation. Moore et al. (2016), also found higher levels of PIP3 in the cilium and hypothesized PIP3 might control AC5/6 function. Live-cell imaging using the FRET PIP3 sensor InPAkt targeted to the cilium via fusion to 5HT6 indeed demonstrated higher local levels of PIP3 compared to the whole cell. Since AC5/6 are inhibited by Ca^2+^, and SAG raises Ca^2+^ levels in the cilium, (Delling et al., 2013), the cilium-targeted Ca^2+^ sensor 5HT6-mCherry-GECO1.0 was used to measure Ca^2+^ after SHh stimulation. Ciliary Ca^2+^ was augmented after SHh activation but cAMP levels were reduced, supporting the hypothesis of Ca^2+^-dependent inhibition of AC5/6 in the cilium. In summary, Moore et al. (2016), were able to show ciliary and cytosolic differences in cAMP signalling, whereby in the cilium, cAMP was upregulated not through GPCR but via PIP3 signalling, and, in contrast to Lesiak et al. and Jiang et al., downregulated through SHh. Moore’s findings do, however, suggest a ciliary cAMP signalosome whereby AC5/6 are regulated by Ca^2+^ rather than Gαs. Such a paradigm is consistent with Choi et al. (2011) findings, where Polycystin regulation of Ca^2+^ signalling modulates AC function at AKAP150 to regulate downstream ciliary cAMP.

cAMP can diffuse freely through the TZ, so it is no surprise that its level ultimately equilibrates in the cilium and cytosol. However, the cAMP pathway does rely on the tight spatio-temporal compartmentalisation of its signal, and far less-isolated cAMP subdomains than the primary cilium have been defined (Surdo et al., 2017; Anton et al., 2022; Subramaniam et al., 2023). For example, at the plasma membrane of cardiac myocytes adrenergic signalling activates PKA to phosphorylate several downstream substrates such as Phospholamban (PLB), Troponin I (TnI), and β2AR, resulting in increased strength of contraction. At the same membrane, Prostaglandin E1 (PGE1) stimulation leads to cytosolic elevation of cAMP with no effect on downstream PKA phosphorylation of these targets or increase in ventricular force generation (Hayes et al., 1980; Di Benedetto et al., 2008). Due to the high surface to volume ratio, one would expect to see in the cilium a higher concentration of cAMP than cytosolic, and Moore et al. have indeed reported increased PKA activity at the cilium (Moore et al., 2016; Arena and Hofer, 2021). The inability to detect differences in cAMP levels between the two compartments may depend on the properties of the cAMP reporters used. cAMP in cells diffuses at a speed in the range of 136–780 µm^2^/s (Agarwal et al., 2016) and the average cilium length is 3–8 µm. Even assuming the slowest cAMP diffusion rate, the highest acquisition rates achievable with FRET imaging while limiting bleaching of the fluorescent signal (typically one image every 3–5 s) may still be too slow to detect swift diffusion of cAMP through the primary cilium. An alternative explanation is that Arl13B may not be the best anchoring protein for detection of compartmentalised ciliary cAMP, as Arl13B localisation in the cilium might be too far away from relevant ciliary cAMP signalosomes, which may be as small as few tens of nanometres (Surdo et al., 2017; Anton et al., 2022).

### Photo-activatable adenylyl cyclases (bPACs)

As mentioned above, targeting of FRET reporters to the cilium via fusion to GPCR has proven challenging. Alternative ciliary-targeting strategies, such as use of ciliary AKAPs, may overcome current limitations, although the identity of these proteins remains largely elusive. Other tools have also been developed, such as photo-activatable ACs (bPACs) as well as Designer Receptors Exclusively Activated by Designer Drugs (DREADDs) and a recent series of elegant experiments used these reagents in combination with FRET imaging to manipulate cAMP production in a more targeted manner.

A ground-breaking study carried out by Truong et al. (2021) used ciliary-bPACs (Stierl et al., 2011) to exclusively generate cAMP within the primary cilium, and showed that this cAMP goes on to strictly affect ciliary, but not cytosolic PKA. Ciliary PKA then exerts different effects on SHh signaling than PKA activated by cytosolic cAMP. Cilium-bPACs were expressed in developing zebrafish embryos and activated using pulsed light to specifically synthesise cAMP at the cilium and not in the cell body. Activation of ciliary bPACs led to somite geometry and neural tube anomalies in the embryos, reminiscent of aberrant SHh signalling. When bPACs localising to the cytosol (cyto-bPAC) were activated, an increase in cAMP was still observed, but embryos did not show developmental defects. Gli:mCherry, which induces mCherry expression upon SHh signal activation, was used alongside cilia-bPAC or cyto-bPAC to confirm that the developmental defects observed upon ciliary cAMP stimulation were indeed SHh related. Only ciliary-bPAC, when exposed to light, attenuated the SHh response. SHh gene expression was also altered via cilia-derived cAMP, but not via cytosolic surges in cAMP (Truong et al., 2021).

Truong et al. (2021) also asked whether basal cAMP levels differed between the two cellular compartments. The cAMP indicator, Pink Flamindo, becomes more fluorescent upon binding to cAMP. (Harada et al., 2017). To test whether cAMP diffuses through the TZ, ciliary Pink Flamindo was used to measure cAMP changes in the cilium upon cyto-bPAC activation. Cyto-bPAC activation enhanced cAMP levels in the cilium, however, surprisingly, not to the same extent as when ciliary-bPACs were triggered. These findings suggest that cAMP can diffuse from the cytosol to the cilium, but cytosolic cAMP does not match the levels of local cAMP production in the organelle. A possible explanation for such observations is that when cytosolic cAMP is stimulated, the second messenger can diffuse from the cell body to the cilioplasm. However, when both ciliary and cytosolic receptors are activated, or if only ciliary GPCRs are stimulated, then the primary cilium generates a significantly concentrated pool of cAMP, with excess second messenger spilling into the cytosol.

DREADDs are designer G_αs_ -coupled receptors which are strictly activated by cognate designer drugs such as clozapine-N-oxide (CNO). Truong et al., used DREADDs to test their hypothesis that ciliary GPCRs regulate SHh signalling and not plasma membrane receptors. Arl13B was used to localise DREADDs to the cilium and the complex tagged with GFP. Activation of cilia-DREADD with CNO, in the presence of SAG, inhibited Gli1 expression, but no attenuation of Gli1 was observed when cytosolic-DREADD was stimulated in the same fashion in NIH/3T3. These results suggest ciliary activation of GPCRs is responsible for regulation of SHh signalling, although they contradict findings by Moore et al. (2016) showing that G_αs_ signalling does not control the SHh pathway.

Truong et al. (2021) also asked whether the ciliary cAMP signal was transmitted through ciliary or cytosolic PKA. Dominant-negative PKA (dnPKA), with no catalytic function, was targeted to either the basal body, the cilium or the cytosol. Cells expressing Gli:mCherry and one of the three dnPKAs demonstrated that only ciliary-dnPKA increased levels of SHh signalling and only ciliary-dnPKA altered levels of the transcription factor Engrailed (EN), which regulates somite patterning (Barresi et al., 2001). These studies support the presence of a fully independent ciliary cAMP signalosome that includes GPCRs, ACs, and PKA and that regulates SHh transcription.

### Ciliary cAMP drives cystogenesis

**FIGURE 5:**
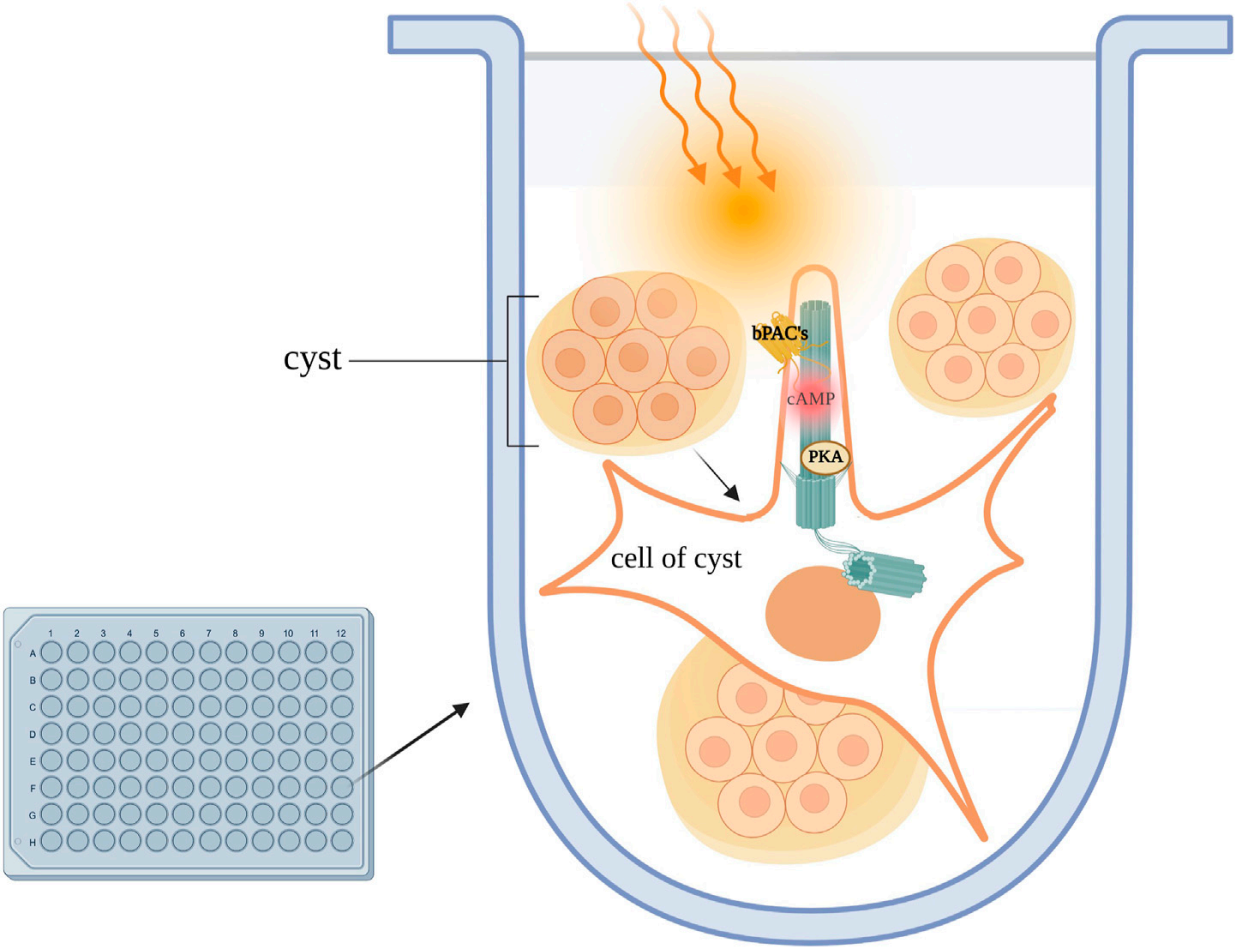
Kidney cells are plated in a 3D culture Matrigel/collagen mix and exposed to light, activating ciliary-bPACs. Enhanced ciliary cAMP synthesis due to AC stimulation leads to activation of ciliary PKA. Ciliary PKA phosphorylates downstream targets which drive cystogenesis in *in-vitro* models of PKD.

The optogenetic approaches described above are powerful tools and promise to provide novel insight that can be exploited for the treatment of ciliopathies. In a recent study, optogenetics was used in combination with RNA sequencing to better characterise the role of ciliary-cAMP signaling in ADPKD. With the aid of ciliary and cytosolic bPACs Hansen et al., 2022 corroborate the previous finding that the primary cilium is a cAMP signaling domain distinct from the cytosol. To address whether ciliary cAMP drives cystogenesis, the group generated a mouse IMCD3 cell line stably expressing ciliary or cytosolic bPAC. When these cells were stimulated with either the universal AC activator, forskolin, or PGE2 in a 3D *in vitro* culture system to emulate the *in vivo* cyst environment, only photo-activation of ciliary-bPAC, and not photo-activation of cytosolic-bPAC led to cyst formation (Figure 5). (Elberg et al., 2012) The group went on to show that ciliary cAMP signalling engages the mTOR pathway to increase cell proliferation, which in turn drives cystogenesis. If a cilium-confined pool of cAMP is the trigger for cyst formation, then selective manipulation of this local pool of second messenger, perhaps via activation of PDEs, may provide a viable strategy to control cyst growth with potentially significantly limited side effects. Isoforms of the PDE4C family had previously been shown to localise to primary cilia and to associate with the PC1/PC2 complex as well as AC5/6, (Choi et al., 2011), and PDE4 inhibition had been shown to be sufficient to induce cystogenesis in MDCK cells as well as primary cells derived from ADPKD patients (Omar et al., 2019). Consistently, inhibition of PDE4 in cells expressing ciliary or cytosolic bPAC IMCD3 induced cyst growth in cilia-bPAC cells in the dark, and exacerbated cyst formation when cells were exposed to light (Hansen et al., 2022). Treatment with a small-molecule activator of PDE4 long-forms indeed abrogated cystogenesis. RNA sequencing of cilia-bPAC versus cytosolic-bPAC stimulated cells revealed that signal transduction, GPCR signalling, metabolism and gene expression pathways were all upregulated selectively in the ciliary-bPAC stimulated cells and CREB was found to be a prominent transcription factor upregulated upon cilia-bPAC stimulation, indicating that the ciliary cAMP signal maintains specificity of downstream target activation (Hansen et al., 2022). Together, these findings suggest ADPKD is driven specifically by excess ciliary cAMP signalling. Additionally, Hansen et al. highlight the role of PDE4 as the main hydrolyser of cAMP in the organelle. The prominent role of PDE4 in the hydrolysis of cAMP in the cilium suggest that pharmacological activation of PDE4 long-form isoforms could be a therapeutic strategy for ADPKD Other PDEs may be involved in the regulation of cAMP at locations that contribute to the pathogenesis of ADPKD. An alternative and more selective approach to pharmacological manipulation of PDEs activity would be to interfere with the interaction of selected relevant PDE isoforms with proteins that anchor the PDE within cAMP signalosomes, including those within the cilium, to achieve a change in cAMP levels only at that specific location and a more targeted functional effect.

## Conclusion

FRET imaging using cilia-targeted reporters alongside the application of optogenetic tools provides strong support to the notion that the cilium can handle cAMP independently of the cytosol. Furthermore, many examples have highlighted how ciliary localization of GPCRs leads to a distinct cAMP response compared to stimulation of the same GPCRs when it is localized to the cell body plasma membrane. These findings indicate that the cilium is a vital center for signaling control, and suggest it may be possible to manipulate selectively cAMP within the cilium to treat ciliopathies such as ADPKD with subcellular precision. However, many questions about the regulation and role of ciliary cAMP remain unanswered. For example, how many cAMP signalosomes exist in the primary cilium and how is their activity coordinated? Why do GPCRs localized to the cilium behave differently when localized to other parts of the plasma membrane? What PDEs control ciliary cAMP from diffusing into the cell body, and could PDE isoforms exclusively localized to the cilium be directly targeted for precision medicine? Could ciliary cAMP signaling be altered without disturbing cytosolic cAMP? With the help of current tools and development of future technology, the full characterization of ciliary cAMP signaling will be possible, with the potential of revealing new insights into the many functions of this mysterious organelle.
